# Mycobiota and Natural Incidence of Aflatoxins, Ochratoxin A, and Citrinin in Indian Spices Confirmed by LC-MS/MS

**DOI:** 10.1155/2015/242486

**Published:** 2015-07-02

**Authors:** Punam Jeswal, Dhiraj Kumar

**Affiliations:** Post-Graduate Department of Biotechnology, A. N. College, Patna, Bihar 800013, India

## Abstract

Nine different Indian spices (red chilli, black pepper, turmeric, coriander, cumin, fennel, caraway, fenugreek, and dry ginger) commonly cultivated and highly used in India were analysed for natural occurrence of toxigenic mycoflora and aflatoxins (AFs), ochratoxin A (OTA), and citrinin (CTN) contamination. *Aspergillus flavus* and *Aspergillus niger* were the most dominant species isolated from all types of spices. Red chilli samples were highly contaminated with aflatoxins (85.4%) followed by dry ginger (77.7%). 56% *Aspergillus flavus* from red chilli and 45% *Aspergillus ochraceus* from black pepper were toxigenic and produced aflatoxins and ochratoxin A, respectively. Qualitative detection and quantitative detection of mycotoxins in spices were analyzed by ELISA and further confirmed by LC-MS/MS. *Penicillium citrinum* produced citrinin in red chilli, black pepper, coriander, cumin, fenugreek, and dry ginger samples. The highest amount of AFs was found in red chilli (219.6 ng/g), OTA was in black pepper (154.1 ng/g), and CTN was in dry ginger samples (85.1 ng/g). The results of this study suggest that the spices are susceptible substrate for growth of mycotoxigenic fungi and further mycotoxin production. This is the first report of natural occurrence of citrinin in black pepper and dry ginger from India.

## 1. Introduction

Spices are cultivated worldwide but India is the largest producer and exporter country in the world [1]. Red chilli, black pepper, turmeric, coriander, cumin, fennel, caraway, fenugreek, and dry ginger are common spices used in Indian cuisine. They provide aroma, color, and flavor in food and are generally used as appetizer to enhance the appetite. They are the herbs, obtained from different parts of plants and rich in antioxidant. These spices have also some medicinal properties and are used widely in ayurvedic medicine [2] and household treatments.

Mycotoxins are the toxic secondary metabolites of fungi produced on wide range of consumable substrates. The most common fungal spices that produce mycotoxins belong to* Aspergillus*,* Penicillium*, and* Fusarium* genera. Some species of these genera have potential to produce different mycotoxins such as aflatoxins, ochratoxin, and citrinin. Aflatoxins are naturally occurring secondary metabolites from some species of* Aspergillus* and they are carcinogenic [3]. OTA and CTN are also produced by some species of* Aspergillus* and* Penicillium*. OTA is hepatotoxic and CTN is nephrotoxic and cooccurrence of these two mycotoxins causes hepatorenal carcinogenesis [4, 5].

Mycotoxin contamination especially aflatoxins contamination in cereals, pulses, oil-seeds, and agricultural products is well known but fragmentary reports are available regarding mycoflora and mycotoxins contamination in spices from India and different part of the world. However these reports are mainly confined to aflatoxins and ochratoxin A contamination [6–8]. This is the first report of CTN in black pepper, cumin, fenugreek, and dry ginger from Bihar.

The present study was conducted to assess the association of toxigenic mycoflora in spices and their mycotoxin producing potentiality. The natural contamination of AFs, OTA, and CTN was also examined in these spices. It has been observed that red chilli, black pepper, coriander, fenugreek, and dry ginger samples were highly susceptible to AFs, OTA, and CTN contamination.

## 2. Material and Methods

### 2.1. Sampling

Nine different Indian spices comprise (55 samples of red chilli, 42 black pepper, 35 turmeric, 30 coriander, 28 cumin, 25 fennel, 25 caraway, 35 fenugreek, and 36 dry ginger) samples. A total 311 samples of different spices were collected from local markets of rural and urban areas of different district of Bihar (state). 100 gm of each sample was kept into sterile brown envelop and stored at 4°C to arrest any mycotoxin formation before analysis.

### 2.2. Screening of Fungi

Samples were randomly placed on the freshly prepared PDA (potato dextrose agar), standard blotter paper and incubated at 28 ± 2°C for 7 days and examined daily. All plates were examined visually and by binocular stereomicroscope and counts were recorded. Fungal colonies of different morphological type were sub-cultured in culture tube containing PDA media. Identification of fungi was carried out by morphological characteristics and followed the taxonomic schemes of Maren [9] for genus* Aspergillus*, Pitt [10] for* Penicillium*, Leslie and Summerell [11] for* Fusarium*, and Crous et al. [12] for other genera.

### 2.3. Assessment of Potentiality of Toxigenic Fungi

*A. flavus*,* A. parasiticus*,* A. ochraceus*,* A. terreus*,* P. citrinum*, and* P. verrucosum* are well known toxigenic fungi to produce aflatoxins, ochratoxin A, and citrinin [13] and they were examined for their mycotoxins producing potentiality. The suspensions of isolated fungi were prepared using 0.5 McFarland standard in normal saline that each mL of saline contains 10^6^ spores [14]. In all cases 50 *μ*L of each suspension was inoculated in 25 mL of freshly prepared broth media (SMKY media for aflatoxins and YES media for ochratoxin A and citrinin) and incubated at 28 ± 2°C for 10 days. When vigorous growth of fungus occurred the medium was filtered with Whatman No. 1 paper and the cultured filtrate was extracted with 10 mL of chloroform. In case of CTN, the culture filtered was acidified with 1N HCL to bring down the pH subsequently; then it was extracted with chloroform. The chloroform extract was evaporated to dryness and residue was dissolved in 1 mL of chloroform and qualitative and quantitative estimations of mycotoxins producing potentiality of fungi were done by the method of Diener and Davis [15] for aflatoxins producing potentiality of* Aspergillus* species. Methods of Schwenk et al. [16] and Davis et al. [17] were followed for CTN and OTA producing potentiality of mycoflora, respectively.

### 2.4. Qualitative and Quantitative Estimation of Mycotoxins by ELISA

Natural occurrence of AFs, OTA, and CTN in spices samples was analyzed by enzyme linked immunosorbent assay (ELISA). Samples were examined by AgraQuant Total Aflatoxin (COKAQ1000) for total AFs and AgraQuant Ochratoxin (COKAQ2000) for OTA from ROMER LAB (ASTRIA) and RIDASCREEN FAST citrinin Assay (6302) for CTN. For qualitative and quantitative estimation of total AFs, OTA and CTN, 20 gm of grinded sample was mixed with 100 mL of 70% methanol and further blended for 3 minutes. The solutions were filtered and the supernatant was collected. 4 mL of extract was transferred through cleanup columns; then the amount of AFs, OTA, and CTN was detected with specific ELISA kits and the optical density was recorded by the ELISA reader using a 450 nm filter with a differential filter of 630 nm. The minimum detected amount of ELISA kit was 4 ng/g for AFs, 2 ng/g for OTA, and 15 ng/g for CTN (as mentioned by the manufacturer of Kits). Standard curve was prepared with standard solution provided with ELISA kits. The optical densities of the samples were compared to the optical density of standards and interpretative results were determined.

### 2.5. Confirmation by LC-MS/MS

Positive samples were further confirmed by LC-MS/MS using Agilent Poroshell 120 EC C18, 2.1 × 100 mm column. 10 gm of grinded sample was mixed with 40 mL of extraction solution containing Acetonitrile : Water (40 : 10, v/v) and vortex vigorously for 5 minute and further shaken gently for another 45 minutes. The solution was filtered through 0.2 *μ* nylon syringe filter. 2 mL of filtrate was taken and dried under fine stream of N_2_ gas further 1 mL of reconstitute solution of Acetonitrile : Water (10 : 40, v/v) was added to prepare sample for LC-MS/MS analysis. 0.5 *μ*L of sample was injected into LC-MS/MS (Agilent 6410) containing the mobile phase of 0.1% formic acid in 5 mM ammonium acetate and methanol [18].

### 2.6. Statistical Analysis

All the samples were analyzed in triplicate. Percent incidence was recorded as the mean value calculated from the number of samples analyzed from triplicate plating. ANOVA test was conducted to determine the differences in mean by one-way ANOVA using SPSS version 12.0 for MS/Windows (SPSS, Inc., Chicago, IL).

## 3. Result and Discussion

### 3.1. Mycobiotic Association

**Figure 1:**
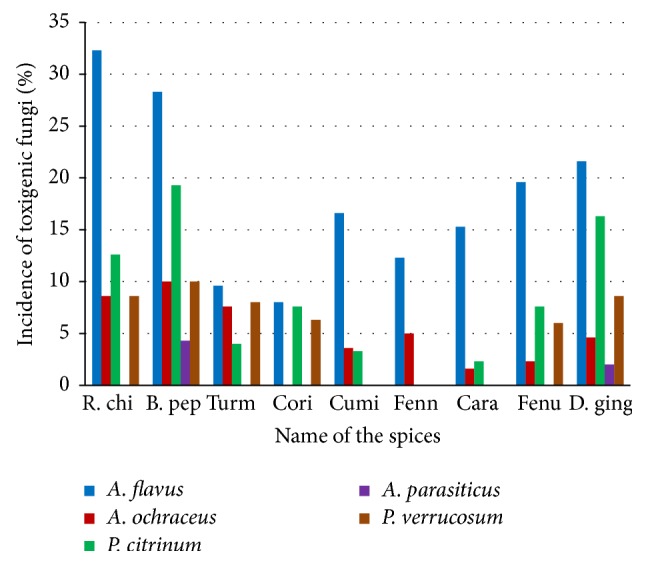
Percent incidence of toxigenic fungal contamination in spices samples.

**Figure 2:**
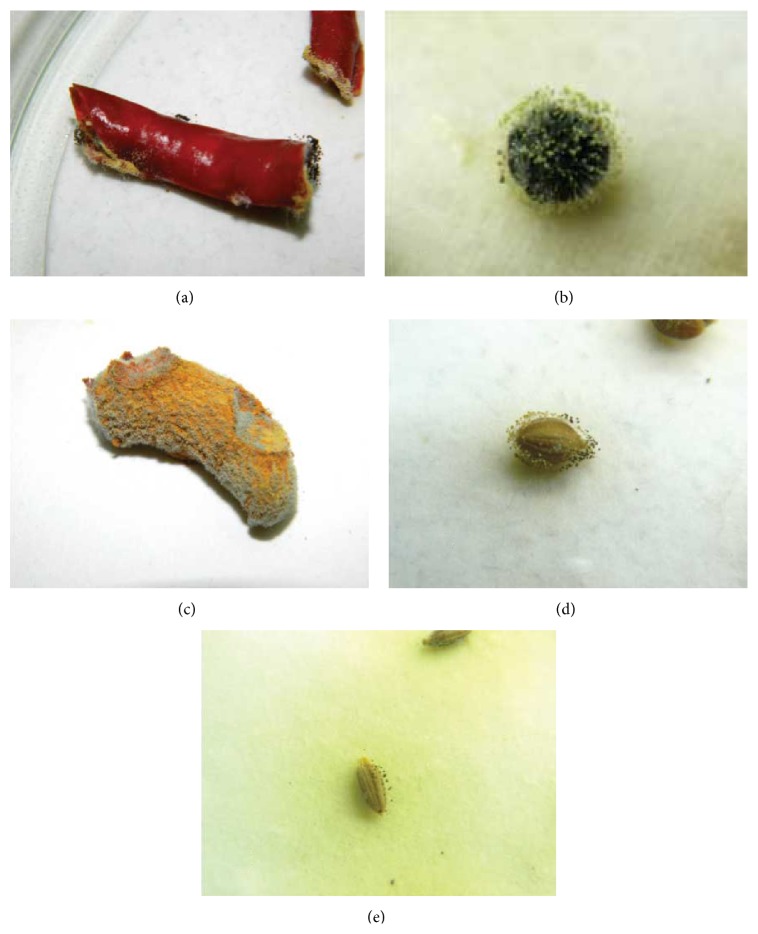
Fungal association of toxigenic fungi in different spices. (a) Red chilli associated with* A. flavus* and* A. niger*, (b)* A. flavus*,* A. parasiticus*,* A. niger*, and the other fungal contamination in black pepper, (c) fungal contamination in turmeric, (d) coriander associatedwith different fungi, and (e) association of* A. flavus* and* A. niger* in cumin.

In our study, prevalence of mycoflora was isolated from spices; total 22 species belong to 7 different fungal genera (Table 1).* Aspergillus* was isolated from all spice samples whereas* Penicillium* were confined only to red chilli, black pepper, turmeric, cumin, coriander caraway fenugreek, and dry ginger samples (Figure 1).* A. flavus* contamination was highest in red chilli (32.3) followed by black pepper (28.3) and dry ginger (21.6) and lowest in coriander (8.0) samples. The present study revealed the wide range of fungal contamination in spices in which* A. flavus* was the most dominant fungal species among all fungi (Figure 2). Recently Rawat et al. [19] have also reported some of these fungi from stored medicinal plant samples. Bokhari [20] has also reported* A. flavus* and* A. niger* contamination from black pepper and green cardamom samples from Saudi Arabia. Moreover, the result from Table 1 also revealed that some of the fungi were only confined to specific spices.* A. alternata*,* A. tamari*, and* C. globosum* species were only confined to red chilli samples whereas* P. citrinum* and* A. ochraceus* were present in all spices except in fennel and coriander, respectively. It may be possible that some of the specific nutrients or essential oil present in these spices can have effect on the growth of specific fungi and further mycotoxin production. Few earlier reports are available regarding inhibitory effects of some spices on the growth of fungi and their mycotoxin production [21, 22]. It has been also observed that red chilli, black pepper, and dry ginger are susceptible substrate for growth of* A. niger*,* A. flavus*,* P. citrinum*,* P. verrucosum*, and* F. moniliforme* whereas coriander, turmeric, cumin, fennel, and fenugreek are mildly resistant.

### 3.2. Toxic Potentiality of Isolated Fungi

Aflatoxins, ochratoxin A, and citrinin producing potentiality of* A. flavus*,* A. parasiticus*,* A. ochraceus*,* A. terreus*,* P. citrinum*, and* P. verrucosum* isolated from spices samples were examined (Table 2). Toxigenic* A. flavus* were isolated from all spices. 56% isolates of* A. flavus* from red chilli samples were highly toxic and produce aflatoxins up to 33.6 *μ*g/L whereas aflatoxins produced by* A. parasiticus* were less potential than* A. flavus*.* A. ochraceus* and* P. verrucosum* produce OTA from all spices, except in cumin. CTN was only produced by* P. citrinum*, up to 18.5 *μ*g/L and it was only confined to red chilli, black pepper, coriander, cumin, fenugreek, and dry ginger samples whereas none of the isolates of* A. terreus* were found toxigenic.

### 3.3. Natural Occurrence of Mycotoxins in Spices

**Figure 3:**
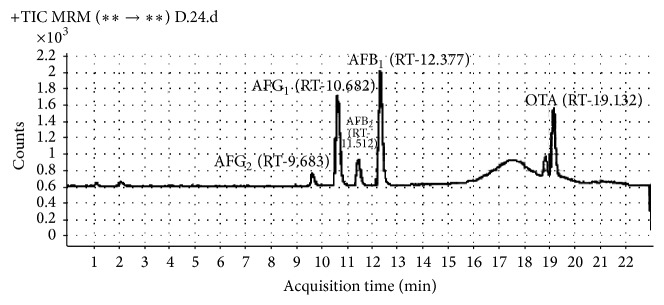
LC-MS/MS chromatogram of AFs (AFB_1_, AFB_2_, AFG_1_, and AFG_2_) and OTA for red chilli sample having maximum contamination.

**Figure 4:**
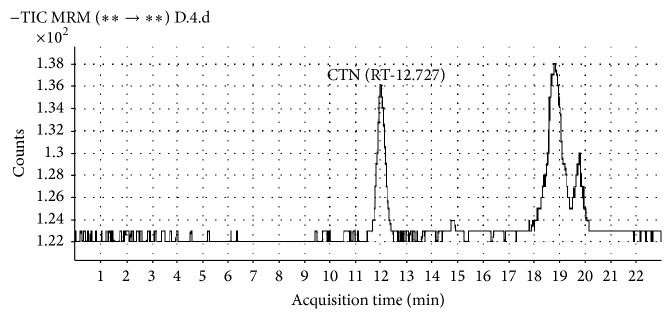
LC-MS/MS chromatogram of CTN for ginger sample having maximum contamination.

AFs (AFB_1_, AFB_2_, AFG_1_, and AFG_2_), OTA, and CTN were detected from different spices (Figures 3 and 4). All 9 types of spice samples were analyzed and it has been observed that some of the samples were only contaminated to aflatoxin B_1_ or B_1_B_2_ or G_1_ or G_1_G_2_ and some were positive to B_1_B_2_G_1_G_2_ whereas OTA and CTN were also detected from these samples (Table 3). 47 out of 55 samples of red chilli were contaminated with aflatoxins in which 31 samples were positive to AFB_1_. Earlier Golge et al. [23] also reported the level of aflatoxins in commercially used Turkish red chilli. CTN contamination was highly observed in dry ginger whereas none of the samples of turmeric, fennel, and caraway were CTN contaminated. OTA were detected from all different spices, except in cumin. Fazekas et al. [24] also reported AFB_1_, AFB_2_, AFG_1_, AFG_2_, and OTA contamination in spices from Hungary. It has been observed that CTN contamination is mainly confined to the dry ginger, red chilli, coriander, and black pepper samples. These samples are highly contaminated with* P. citrinum* or* P. verrucosum* or both and they were known to produce CTN on substrate. So, it may be possible that dry ginger, red chilli, coriander, and black pepper are susceptible for* P. citrinum* and* P. verrucosum* growth and further mycotoxin productions.

**Figure 5:**
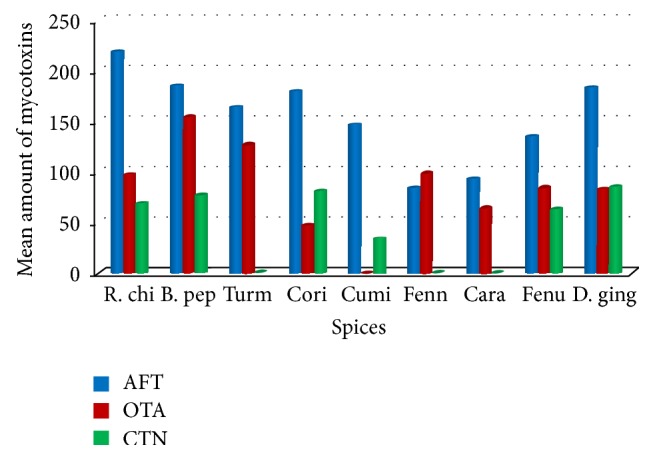
Amount of total aflatoxins, ochratoxin A, and citrinin in different spices.

Natural occurrence of AFs, OTA, and CTN in spices has been shown in Table 4. All spices were contaminated with AFs and detected amount is maximum compared to OTA and CTN except in fennel. Highest amount of AFs was recorded in red chilli samples (219.6 ng/g) and OTA contamination was maximum in black pepper (154.1 ng/g). Earlier Jalili and Jinap [25] have reported that 65% of chilli samples were contaminated with AFs level in the range of 0.2–79.7 ng/g and 81.25% of samples were positive to OTA in the range of 0.2–101.2 ng/g. Ozbey and Kabak [26] have also reported 30.4% AFs contamination and 17.4% of OTA contamination in black pepper powder. In present investigation, only 30% of coriander samples were positive to OTA and none of the cumin samples were positive. It may be due to essential oil (cuminaldehyde) of the cumin which inhibited the growth of OTA producing fungi (*A. ochraceus* and* P. verrucosum*) and OTA production. Earlier, Hua et al. [27] have reported that essential oil of cinnamon inhibits the growth of* A. ochraceus* and ochratoxin A production. Ferreira et al. [28] have reported that turmeric has the inhibitory effect on the growth of* A. flavus* and mycotoxin production but in our report 68.5% of turmeric was AFs contaminated and 57.1% was OTA contaminated with the detectable amount of 163.8 ng/g and 125 ng/g, respectively (Figure 5).

## 4. Conclusion

On the basis of the present study, it may be concluded that the red chilli, black pepper, and dry ginger are susceptible substrate for fungal growth and subsequent mycotoxin productions. All 9 types of spices were contaminated with AFs. This is the first report of CTN contamination in black pepper, cumin, fenugreek, and dry ginger from Bihar state (India). Red chilli, black pepper, and dry ginger are the most contaminated spices in which AFs, OTA, and CTN were present in high concentration. Fennel, caraway, and cumin are the spices which can be considered a bit resistant to mycotoxigenic fungi and mycotoxin contamination. Further research is needed to isolate the active ingredient or the essential oil of these spices, which plays a vital role in the growth of toxigenic fungi and further toxin production. It is very important to care in processing, handling, transportation, and modification in storage system to reduce the production of hazardous mycotoxins in spices.

## Figures and Tables

**Table 1:** Percent incidence of fungal mycoflora isolated from different spices.

Fungal species	Name of the spices								
	R. chi^a^	B. pep^b^	Turm^c^	Cori^d^	Cumi^e^	Fenn^f^	Cara^g^	Fenu^h^	D. ging^i^
*Alternaria alternata *	2.3	—	—	—	—	—	—	—	—
*Aspergillus parasiticus *	—	4.3	—	—	—	—	—	—	2.0
*Aspergillus oryzae *	—	8.3	—	—	1.6	—	3.3	—	1.3
*Aspergillus tamarii *	5.3	—	—	—	—	—	—	—	—
*Aspergillus niger *	15.3	19.6	8.0	10.6	9.6	5.3	4.3	2.6	9.0
*Aspergillus flavus *	32.3	28.3	9.6	8.0	16.6	12.3	15.3	19.6	21.6
*Aspergillus ochraceus *	8.6	10.0	7.6	—	3.6	5.0	1.6	2.3	4.6
*Aspergillus versicolor *	11.4	7.65	—	—	—	—	—	—	—
*Aspergillus fumigatus *	—	—	—	2.0	—	—	—	—	4.6
*Aspergillus terreus *	—	6.3	—	—	2.6	1.3	—	—	2.6
*Aspergillus sydowii *	—	—	—	2.6	—	—	4.0	2.6	—
*Penicillium citrinum *	12.6	19.3	4.0	7.6	3.3	—	2.3	7.6	16.3
*Penicillium islandicum *	—	—	—	—	—	—	1.6	—	—
*Penicillium verrucosum *	8.6	10.0	8.0	6.3	—	—	—	6.0	8.6
*Penicillium purpurogenum *	—	1.3	—	—	—	—	—	—	2.6
*Penicillium cyclopium *	—	—	—	—	—	—	1.6	—	—
*Fusarium oxysporum *	—	5.6	5.3	—	6.6	—	3.3	—	2.3
*Fusarium moniliforme *	6.3	9.6	4.6	—	—	3.0	—	1.6	3.0
*Chaetomium globosum *	1.4	—	—	—	—	—	—	—	—
*Rhizopus nigricans *	—	6.3	—	4.3	2.5	—	—	—	—
*Rhizopus oryzae *	4.3	6.6	—	—	2.6	2.3	—	4.0	2.6
*Mucor hiemalis *	7.0	3.0	—	2.6	—	1.6	—	—	—

^a^Red chilli, ^b^black pepper, ^c^ turmeric, ^d^coriander, ^e^cumin, ^f^fennel, ^g^caraway, ^h^fenugreek, and ^i^dry ginger.

**Table 2:** Mycotoxin producing potentiality of toxigenic fungi from different spices.

Sn	Spice	Fungi examined	% tox^a^	Mycotoxin detected	Potential range (*µ*g/L)
1	R. chi	*A. flavus *	56.0	AFs	4.3–33.6
		*A. ochraceus *	41.6	OTA	2.8–18.6
		*P. citrinum *	31.2	CTN	15.6–28.3
		*P. verrucosum *	30.0	OTA	3.5–8.6

2	B. pep	*A. flavus *	45.4	AFs	6.3–26.4
		*A. parasiticus *	26.6	AFs	4.8–9.6
		*A. ochraceus *	45.0	OTA	5.4–22.6
		*A. terreus *	0	—	—
		*P. citrinum *	44.4	CTN	15.4–20.5
		*P. verrucosum *	25.0	OTA	4.6–13.8

3	Turm	*A. flavus *	20.0	AFs	4.4–11.3
		*A. ochraceus *	40.0	OTA	2.1–18.9
		*P. citrinum *	0	—	—
		*P. verrucosum *	16.6	OTA	3.7–6.1

4	Cori	*A. flavus *	38.8	AFs	5.2–13.7
		*P. citrinum *	26.6	CTN	18.7–20.9
		*P. verrucosum *	20.0	OTA	6.8–16.1

5	Cumi	*A. flavus *	46.6	AFs	5.8–18.2
		*A. ochraceus *	0	—	—
		*A. terreus *	0	—	—
		*P. citrinum *	13.3	CTN	16.0–18.5

6	Fenn	*A. flavus *	45.0	AFs	8.7–24.3
		*A. ochraceus *	38.8	OTA	6.5–8.4
		*A. terreus *	0	—	—

7	Cara	*A. flavus *	40.9	AFs	6.4–16.7
		*A. ochraceus *	22.2	OTA	6.1–15.4
		*P. citrinum *	0	—	—

8	Fenu	*A. flavus *	35.0	AFs	6.4–19.7
		*A. ochraceus *	40.0	OTA	3.5–20.1
		*P. citrinum *	20.0	CTN	17.2–19.2
		*P. verrucosum *	0	—	—

9	D. ging	*A. flavus *	44.0	AFs	5.8–24.2
		*A. parasiticus *	27.7	AFs	6.4–8.1
		*A. ochraceus *	33.3	OTA	3.1–16.7
		*A. terreus *	0	—	—
		*P. citrinum *	38.8	CTN	16.4–22.3
		*P. verrucosum *	30.0	OTA	2.4–8.7

^a^Percentage of toxigenic isolates of fungi.

**Table 3:** Number of different spice samples contaminated with aflatoxins, ochratoxin A, and citrinin.

Spices	Number of samples analyzed	Number of samples with different mycotoxins contamination							
		Aflatoxins						OTA	CTN
		B_1_	G_1_	B_1_B_2_	G_1_G_2_	B_1_B_2_G_1_G_2_	Total		
Red chilli	55	31	2	9	1	4	47	40	26
Black pepper	42	19	1	5	2	5	32	33	19
Turmeric	35	10	0	7	3	4	24	20	0
Coriander	30	15	0	4	1	2	22	09	12
Cumin	28	12	2	3	0	1	18	0	6
Fennel	25	08	0	4	1	1	14	14	0
Caraway	25	08	1	3	0	1	13	12	0
Fenugreek	35	15	0	3	2	3	23	18	13
Dry ginger	36	16	2	7	0	3	28	20	16

**Table 4:** Natural occurrence and amount of aflatoxins, ochratoxin A, and citrinin detected in different ranges from the spices.

Mycotoxins	Spices	N.S.A^a^	Number of samples present in between different ranges (ng/g)							Amount (ng/g)	CV^d^	% cont^e^
			N.D^b^	LDL^c^–100	101–200	201–300	301–400	401–500	501–≤	Mean ± S.E		
AFs	R. chi	55	8	10	7	8	13	7	2	219.6 ± 21.3	0.7	85.4
	B. pep	42	10	4	3	14	10	1	0	185.0 ± 22.0	0.7	76.1
	Turm	35	11	4	4	6	10	0	0	163.8 ± 25.7	0.9	68.5
	Cori	30	8	2	5	7	6	2	0	179.5 ± 27.2	0.8	73.3
	Cumi	28	10	2	6	3	5	2	0	146.8 ± 28.7	1.0	64.2
	Fenn	25	11	4	5	5	0	0	0	84.1 ± 20.2	1.2	56.0
	Cara	25	12	4	2	6	1	0	0	92.7 ± 23.3	1.2	52.0
	fenu	35	12	6	7	10	0	0	0	135.4 ± 24.8	1.0	65.7
	D. ging	36	8	2	12	7	3	2	2	183.6 ± 25.0	0.8	77.7

OTA	R. chi	55	15	14	22	2	2	0	0	97.1 ± 12.8	0.9	72.7
	B. pep	42	9	7	9	12	5	0	0	154.1 ± 19.3	0.8	78.5
	Turm	35	15	3	5	7	3	2	0	125.9 ± 24.0	1.1	57.1
	Cori	30	21	4	0	4	1	0	0	47.6 ± 17.2	1.9	30.0
	Cumi	30	30	0	0	0	0	0	0	0	0	0
	Fenn	25	11	3	5	4	2	0	0	98.1 ± 22.6	1.1	56.0
	Cara	25	13	6	2	4	0	0	0	63.2 ± 18.9	1.4	48.0
	fenu	35	17	5	6	6	1	0	0	83.2 ± 17.6	1.2	51.4
	D. ging	36	16	6	7	2	3	0	0	82.8 ± 19.0	1.3	55.5

CTN	R. chi	55	29	8	12	5	1	0	0	69.0 ± 12.5	1.3	47.2
	B. pep	42	23	7	6	3	2	1	0	76.9 ± 17.8	1.5	45.2
	Turm	35	35	0	0	0	0	0	0	0	0	0
	Cori	30	18	3	2	4	2	1	0	81.0 ± 23.0	1.5	40.0
	Cumi	28	22	1	4	0	1	0	0	33.9 ± 14.7	2.3	21.4
	Fenn	25	25	0	0	0	0	0	0	0	0	0
	Cara	25	25	0	0	0	0	0	0	0	0	0
	fenu	35	22	3	5	4	1	0	0	63.1 ± 17.2	1.6	37.1
	D. ging	36	20	3	7	2	4	0	0	85.1 ± 19.4	1.3	44.4

^a^Number of samples analyzed, ^b^not detected, ^c^lowest detectable level of ELISA Kit (4 ng/g for AFs, 2 ng/g for OTA, 15 ng/g for CTN), ^d^coefficient of variation, and ^e^percent contamination.

## References

[1] Power S. R. (2013). Present scenario of Indian spice industry and its trend in production and export. *Asian Journal of Management*.

[2] Sherman P. W., Billing J. (1999). Darwinian gastronomy: why we use spices: spices taste good because they are good for us. *BioScience*.

[3] Qureshi H., Ali S. S., Iqbal M., Siddiqui A. A., Khan N. A., Hamin S. S. (2014). Aflatoxins and hepatitis B, C viral associated hepatocarcinogenesis. *Journal of Cell Science & Therapy*.

[4] Jeswal P. (1995). Cumulative effect of Ochratoxin A and citrinin on induction of hepatorenal carcinogenesis in mice (*Mus musculus*). *Biomedical Letters*.

[5] Wichmann G., Herbarth O., Lehmann I. (2002). The mycotoxins citrinin, gliotoxin, and patulin affect interferon-*γ* rather than interleukin-4 production in human blood cells. *Environmental Toxicology*.

[6] Ath-Har M. A., Prakash H. S., Shetty H. S. (1988). Mycoflora of Indian spices with special reference to aflatoxigenic producing isolates of *Aspergillus flavus*. *Indian Journal of Microbiology*.

[7] Zaied C., Abid S., Bouaziz C., Chouchane S., Jomaa M., Bacha H. (2010). Ochratoxin A levels in spices and dried nuts consumed in Tunisia. *Food Additives and Contaminants: Part B Surveillance*.

[8] Tosun H., Arslan R. (2013). Determination of aflatoxin B1 levels in organic spices and herbs. *The Scientific World Journal*.

[9] Maren A. K. (2002). *Identification of Common Aspergillus Species*.

[10] Pitt J. I. (1988). *A Laboratory Guide to Common Penicillium Species*.

[11] Leslie J. F., Summerell B. A. (2006). *The Fusarium Laboratory Manual*.

[12] Crous P. W., Verkley G. D. M., Groenewald J. Z., Samson R. A. (2009). *Fungal Diversity*.

[13] Pitt J. I. (2000). Toxigenic fungi and mycotoxins. *British Medical Bulletin*.

[14] Pfaller M. A., Burmeister L., Bartlett M. S., Rinaldi M. G. (1988). Multicenter evaluation of four methods of yeast inoculum preparation. *Journal of Clinical Microbiology*.

[15] Diener U. L., Davis N. D. (1966). Aflatoxin production by isolates of *Aspergillus flavus*. *Phytopathology*.

[16] Schwenk E., Alexander G. J., Gold A. M., Stevens D. F. (1958). Biogenesis of citrinin. *The Journal of Biological Chemistry*.

[17] Davis N. D., Sansing G. A., Ellenburg T. V., Diener U. L. (1972). Medium-scale production and purification of ochratoxin A, a metabolite of *Aspergillus ochraceus*. *Applied Microbiology*.

[18] Zheng R., Xu H., Wang W., Zhan R., Chen W. (2014). Simultaneous determination of aflatoxin B_1_, B_2_, G_1_, G_2_, ochratoxin A, and sterigmatocystin in traditional Chinese medicines by LC-MS-MS. *Analytical and Bioanalytical Chemistry*.

[19] Rawat A., Mahajan S., Gupta A., Agnihotri R. K., Wahi N., Sharma R. (2014). Detection of toxigenic fungi and mycotoxins in some stored medicinal plant samples. *International Journal of Applied Sciences and Biotechnology*.

[20] Bokhari F. M. (2007). Spices mycobiota and mycotoxins available in Saudi Arabia and their abilities to inhibit growth of some toxigenic fungi. *Mycobiology*.

[21] Tian J., Ban X., Zeng H., He J., Huang B., Wang Y. (2011). Chemical composition and antifungal activity of essential oil from *Cicuta virosa* L. var. *latisecta* Celak. *International Journal of Food Microbiology*.

[22] Mishra A. K., Mishra A., Kehri H. K., Sharma B., Pandey A. K. (2009). Inhibitory activity of Indian spice plant *Cinnamomum zeylanicum* extracts against *Alternaria solani* and *Curvularia lunata*, the pathogenic dematiaceous moulds. *Annals of Clinical Microbiology and Antimicrobials*.

[23] Golge O., Hepsag F., Kabak B. (2013). Incidence and level of aflatoxin contamination in chilli commercialised in Turkey. *Food Control*.

[24] Fazekas B., Tar A., Kovács M. (2005). Aflatoxin and ochratoxin A content of spices in Hungary. *Food Additives and Contaminants*.

[25] Jalili M., Jinap S. (2012). Natural occurrence of aflatoxins and ochratoxin A in commercial dried chili. *Food Control*.

[26] Ozbey F., Kabak B. (2012). Natural co-occurrence of aflatoxins and ochratoxin A in spices. *Food Control*.

[27] Hua H., Xing F., Selvaraj J. N. (2014). Inhibitory effect of essential oils on *Aspergillus ochraceus* growth and ochratoxin a production. *PLoS ONE*.

[28] Ferreira F. D., Mossini S. A. G., Ferreira F. M. D. (2013). The inhibitory effects of *Curcuma longa* L. essential oil and curcumin on *Aspergillus flavus* link growth and morphology. *The Scientific World Journal*.

